# Monitoring Progression in Hypertensive Patients with Dyslipidemia Using Optical Coherence Tomography Angiography: Can A.I. Be Improved?

**DOI:** 10.3390/jcm13247584

**Published:** 2024-12-13

**Authors:** Irina Cristina Barca, Vasile Potop, Stefan Sorin Arama

**Affiliations:** 1 Ophthalmology Department, “Carol Davila” University of Medicine and Pharmacy, 050474 Bucharest, Romania; vasile.potop@umfcd.ro; 2 Physio-Pathology and Immunology Department, “Carol Davila” University of Medicine and Pharmacy, 050474 Bucharest, Romania; sorin.arama@umfcd.ro

**Keywords:** optical coherence tomography angiography, A.I., capillary non-perfusion, dyslipidemia, hypertensive retinopathy

## Abstract

**Background**: With the development of artificial intelligence (A.I.), the optical coherence tomography angiography (OCTA) analysis of progression in hypertensive retinopathy could be improved. Our purpose was to use the OCTA to study the effect of uncontrolled dyslipidemia and hypertensive retinopathy on the retinal microvasculature and to identify a potential software update of the A.I. secondary to the OCTA analysis. By using our most relevant data, the A.I. software can be upgraded by introducing new mathematic formulas between the OCTA parameters and the lipid level. **Methods**: We performed a prospective cohort study on 154 eyes of participants from Eastern Europe. We used a standardized protocol to collect data on past medical history of dyslipidemia and hypertension and OCTA to measure retinal vascular parameters. **Results**: The average age of the participants was 56.9 ± 9.1, with a minimum of 34 and a maximum of 82 and with a higher percentage of males: 55.8%. Statistically significant correlations were found for total cholesterol and skeleton total (r = −0.249; *p* = 0.029), foveal avascular zone (FAZ), circularity and low-density lipoprotein (LDL) (r = 0.313; *p* = 0.006), non-flow area (NFA) and LDL (r = 0.233; *p* = 0.042), and vascular flow area (VFA) and LDL (r = −0.354; *p* = 0.002). **Conclusions**: Subjects with dyslipidemia and progressive hypertensive retinopathy had a reduction in microvascular density and vascular flow, a focal capillary non-perfusion, and an increased FAZ. Thus, by improving the A.I. system, our research aims to provide better OCTA monitoring, which could help in the early-stage detection of progression and development of A.I. screening programs, leading to increased efficiency in diagnosing patients.

## 1. Introduction

According to reports from the World Health Organization (WHO), in March 2023, an estimated 1.28 billion adults aged 30–79 years worldwide had hypertension, with most (two-thirds) living in low- and middle-income countries. An estimated 46% of adults with hypertension are unaware that they have the condition, less than half of adults (42%) with hypertension are diagnosed and treated, and approximately one in five adults (21%) have it under control. Hypertension is a major cause of premature death worldwide and is a major risk factor for stroke, myocardial infarction, vascular disease, and chronic kidney disease. Dyslipidemia results in abnormal levels of lipids in the blood that can also increase the risk of cardiovascular diseases [1]. Dyslipidemia is classified into two types: primary and secondary. Primary dyslipidemia is inherited and caused by genetic mutations that affect lipid metabolism, while secondary dyslipidemia is acquired and caused by lifestyle factors or other medical conditions that alter lipid levels. Both types are associated with hypertension. The management of dyslipidemias is an essential and integral part of cardiovascular disease prevention [2].

Chronic high blood pressure or previous episodes of high blood pressure can affect retinal microcirculatory structure and function [3].

There have been many previous studies on the association between hypertensive retinopathy and hypertension [4,5,6,7,8,9,10].

The prevalence rate of retinopathy in patients with mild hypertension is approximately 25%, with the moderate form of the disease is 34%, and with the severe form is 84.6%. Of the patients with hypertensive retinopathy, 42.3% have a grade I, 20% a grade II, and 2.35% a grade III ocular pathology [5].

Hypertensive retinopathy is related to blood pressure variability (BPV); the degree of BPV and retinopathy in hypertensive patients can be used to track the progression of hypertension-mediated organ damage (HOMD), and the correction of BPV may help treat or postpone the progression of HOMD. Hypertension is associated with reduced retinal vessel density and an increased foveal avascular zone (FAZ), especially in the deep venous plexus, as seen on optical coherence tomography angiography (OCTA) [11].

Patients with hypertensive retinopathy show a reduction in the retinal vascular density, superficial and deep vascular plexus (SVP and DVP), ganglion cell–internal boundary membrane complex density (GCL-ILM), and retinal nerve fiber layer (RNFL) thickness [12].

The classic retinal changes in chronic hypertensive retinopathy include arteriolar narrowing and tortuosity, arteriovenous crossing changes, retinal hemorrhages, cotton-wool spots, hard exudates, papilledema. The malignant stage of hypertensive retinopathy represents a medical emergency, often associated with markedly elevated blood pressure and with the risk of organ damage [8,13].

Compared to invasive fundus fluorescein angiography (FFA), OCTA is a new, rapid, easily performed, and non-invasive 3D imaging modality, allowing healthcare professionals to study the microvasculature of the retinal and choroidal layers in large populations of patients with dyslipidemia and hypertensive retinopathy [14]. The retina and choroid are two distinct vascular beds. Using 3D OCTA, distinct visualizations of these two circulations can be obtained. In healthy eyes, retinal circulation is located between the internal limiting membrane (ILM) and the outer plexiform layer (OPL). The choroidal circulation is located beneath Bruch’s membrane (BM). The vitreous (anterior to the ILM) and outer retina (between OPL and Bruch’s membrane) are avascular structures [14]. OCTA allows for quantitative assessment of the retinal microvasculature, providing metrics such as vessel density and vessel caliber. These measurements help in tracking hypertensive retinopathy progression in patients with dyslipidemia and assessing the efficacy of treatment interventions over time [9,15].

OCTA changes in hypertensive retinopathy include microvascular density reduction, focal capillary non-perfusion, increased FAZ, vascular tortuosity and irregularities, and choroidal thickness alterations [3,9,15,16,17].

The aim of our current study was to measure FAZ, non-flow area (NFA), vascular flow area (VFA), retinal vascular density, and perfusion by using OCTA in patients with chronic hypertension and secondary dyslipidemia, identifying the potential OCTA parameters to be analyzed using A.I. We hypothesize that OCTA will show subclinical changes in retinal microvasculature in the superficial and deep plexi, i.e., changes related to secondary dyslipidemia and progression of hypertensive retinopathy.

## 2. Materials and Methods

### 2.1. Study Participants

The Ethics Committee approved the study in December 2022. Patients were selected for inclusion in this study by the ophthalmologist and referred to the ophthalmologist by a cardiologist, general practitioner, or internist.

We performed a prospective cohort study including 77 participants with dyslipidemia and systemic hypertension from Eastern Europe, Romania. From 2022 to 2024, 154 eyes were eligible for the final analysis presented in this study. All participants underwent ophthalmological examinations, blood testing, blood pressure monitoring, and body mass index (BMI) calculation according to protocol.

For the purpose of this study, we selected subjects diagnosed with chronic hypertension and secondary dyslipidemia (based on medical history and records) as well as undiagnosed cases by determining blood pressure, performing blood tests and fundoscopy, and by referring patients to a cardiologist and internist for an accurate diagnosis.

We selected hypertensive patients with consistently high values in all measurements, including patients using and not using antihypertensive treatments at the time of their first visit. Subjects with normalized blood pressure during follow-ups were not excluded from the study.

Patients with chronic hypertensive retinopathy were included in the study based on fundoscopic examination. We selected grade I–II of hypertensive retinopathy (mild) according to the Keith–Wagener–Barker or Mitchell–Wong classification system and no other types of retinopathy based on fundoscopy [5,18]. Patients with the following pathologies were excluded by using fundoscopy and by scanning the optic nerve and retina in both eyes using OCTA: hypertensive retinopathy grade III (moderate) and IV (severe) according to the Keith–Wagener–Barker or Mitchell–Wong classification system, glaucoma, diabetic retinopathy, maculopathy, central serous retinopathy, pigment epithelial detachments, epiretinal membranes, cystoid macula edema, vitreomacular traction, and other significant co-morbidities that could affect retinal vasculature.

In our study, we included participants with newly diagnosed dyslipidemia based on blood testing performed at the time of the first visit and diagnosed before referral by the internist or the cardiologist.

Systolic and diastolic blood pressure was measured twice per month. Blood pressure was taken with an automatic blood pressure monitor (Hartmann Tensoval Comfort, PAUL HARTMANN AG, 89522 Heidenheim, Germany). Three readings were taken 5 min apart, before exercise or at least 30 min after exercise. Hypertension was defined as present in participants with elevated blood pressure at the time of examination (SPB ≥ 130 mmHg or DBP ≥ 80 mmHg), according to American Heart Association criteria [2,16]. Undiagnosed cases were defined to be hypertensive if their systolic blood pressure was more than or equal to 140 mmHg or diastolic blood pressure more than or equal to 90 mmHg or if there was a self-reported history of self-measured blood pressure above the same values.

Height, body weight, and BMI were calculated every 3 months. Height was measured using a portable stadiometer (Seca Model 217; Seca, Hamburg, Germany). Body weight was determined using a calibrated mechanical column scale with eye-level beam (Seca 700; Seca, Hamburg, Germany). BMI was calculated by dividing body weight (kg) by the square of height (m^2^). The age and sex were also noted. Recording of the smoking habit was also performed. Smoking was assessed with the single categorized question: “Are you a current smoker?”.

Glycemia and HbA1c were determined in order to rule out subjects with diabetes and/or undiagnosed diabetic retinopathy. Glycemia, glycated hemoglobin (HbA1c), total cholesterol, HDL, LDL, and triglycerides were measured every 3 months using the same laboratory equipment. Non-fasting blood samples were used to assess plasma lipid profiles. Measurements were performed on all patients with or without statin use at the time of the enrollment. Patients with normalized plasma lipid values after receiving statin treatment during follow-ups were not excluded from the study.

Each participant underwent an ophthalmic examination including refraction measured with Auto Kerato-Refractometer (Topcon, Model KR-800, Tokyo, Japan) and determination of intraocular pressure with Goldmann Tonometer, fundoscopy, and OCTA. All refractive errors were included. All subjects had a normal intraocular pressure ranging from 10 to 21 mmHg. Examination of the retina using a Volk 90D and a 78D power lens (Volk, Mentor, OH, USA) was performed quarterly in order to stage hypertensive retinopathy and to include or exclude participants in our study.

OCTA was performed on both eyes, on all patients, every 3 months, with the assessment of FAZ, NFA, VFA, retinal vascular density, and perfusion on the OCTA. Eyes with poor-quality images or a poor signal strength were excluded.

### 2.2. Imaging Protocol

OCTA of the macula was performed using Angio-OCT Optopol Revo NX 130 (OPTOPOL Technology, Zawiercie, Poland) (Angiography module including a Retina Angiography 6 × 6 mm scan program and OCTA mosaic program to merge together 3 × 3 or 6 × 6 mm scans in order to create high-resolution mosaics of a larger area, iTracking™ technology, Motion Correction Technology™ and Projection Artefact Removal algorithm to minimize artifacts). The available vascular layers identified by the OCTA can be selected from the following list: retina, vitreous, superficial capillary plexus, superficial vascular plexus (SVC), radial peripapillary capillary plexus (RPCP), deep capillary plexus, deep vascular plexus (DVC), intermediate capillary plexus (ICP), outer retina layers, choriocapillaris, and choroidal vessels, with depth coded.

For the purpose of this study, 6.0 × 6.0 mm scans were performed, centered on the fovea of the right eye and left eye from each participant. The software (Version 11.0.4 REVO NX Device SN: 1560658/16) was used to analyze the superficial layer (with the offset of inner limiting membrane (ILM) 0 µm, inner plexiform layer/ inner nuclear layer (IPL/INL) 15 µm), the deep layer (with the offset of IPL/INL—15 µm, IPL/INL—70 µm), and the choriocapillaris (with the offset of Bruch’s membrane (BM) top BM 30 µm, bottom BM 45 µm).

The software was also used to assess the FAZ, NFA, VFA, and the retinal vascular density and skeleton. These parameters were derived from an En face angiogram. The FAZ, NFA, and VFA areas were detected and measured using the semi-auto area tool of the software. As the semi-automated measurement was noted to be inaccurate in a number of scans, manual measurements were performed. The calculated areas of the following parameters are provided: FAZ area in mm^2^, FAZ perimeter in mm, circularity (ratio between the measured perimeter and the perimeter of a circular area of the same size), NFA in mm^2^, VFA area in mm^2^, and VFA flow area in mm^2^. Quantification provided the quantification of vasculature in specific sectors and a heat map corresponding to the vasculature.

Density display is defined as the total area of perfused vasculature per unit area in a region of measurement. This metric is calculated by summing up the number of pixels that contain perfused vasculature and dividing the sum by the total number of pixels in the considered region. The result is an unitless number ranging from 0 (no perfusion) to 1 (fully perfused) (mm^2^/mm^2^).

Skeleton display is defined as the total area of skeletonized vasculature per unit area in a region of measurement. Skeletonization performs thinning of all vessels down to 1 pixel width and thus makes analysis more sensitive to small vasculature (as the large vessels lose more area than the thin ones in the skeletonization process). This metric is calculated by summing up the number of pixels that represent the skeleton of the vasculature and dividing the sum by the total number of pixels in the considered region. The result is an unitless number ranging from 0 (no perfusion) to 1 (mm^2^/mm^2^).

Both density and skeleton display can detect abnormal vasculature and provide repeatable quantitative results equally in normal and diseased eyes. Measurement zones for the available 6 mm width retina scans are total, superior, inferior, center, inner, superior inner, inferior inner, outer, superior outer, inferior outer, and the Early Treatment of Diabetic Retinopathy Study (ETDRS) grid.

**Figure 1:**
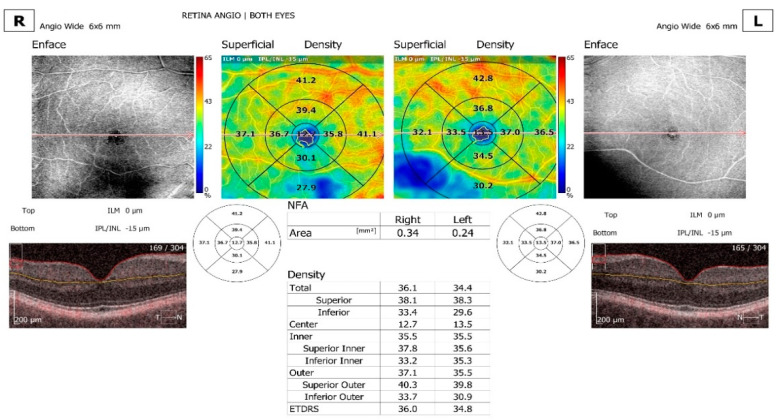
En face OCTA of the superficial vascular plexus with calculated NFA area, comparing right eye and left eye and calculated total, center, inner and outer density. OCT showing that the superficial plexus extends from the inner limiting membrane (top) (0 µm) to the inner plexiform layer/inner nuclear layer (bottom) (IPL/INL—15 µm). ETDRS grid is also available, comparing right eye and left eye.

**Figure 2:**
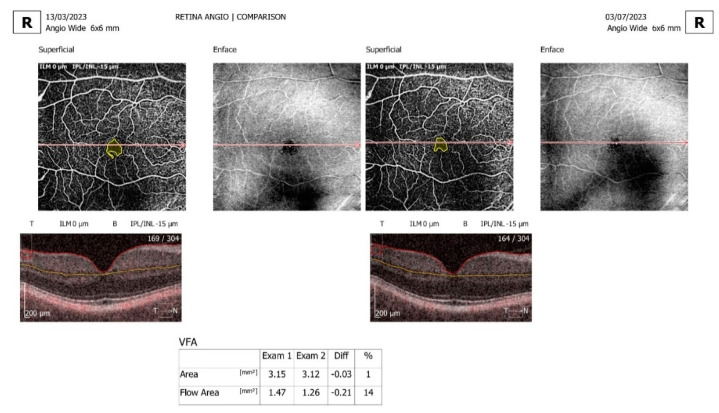
En face OCTA of the superficial vascular plexus with calculated VFA area and flow area, comparing right eye examination number 1 and examination number 2, with the difference between them measured in mm^2^ and percentage.

**Figure 3:**
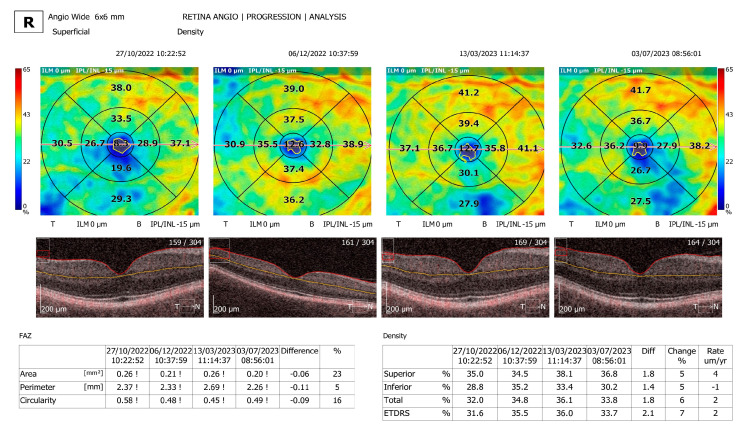
Retina Angiography progression analysis program comparing four scans. FAZ area, perimeter, and circularity were measured and compared. Superior, inferior, total density, and ETDRS grid are shown in a table in the right bottom corner of the OCTA, with calculated difference and change rate in µm/year between the four scans.

Comparison and progression view screens were used. The comparison view screen shows the analysis results, comparing two examinations of one eye on the same side in the same scan mode but from different dates. The comparison view is used to observe follow-up changes in the eye structure. The software automatically selects outermost examinations (the oldest and the newest) in order to compare them. The progression view screen shows the analysis results comparing four examinations, all performed on the same side in the same scan mode and on the same size of scanning area, arranged in a time sequence. Eyes with a significant artifact that obscured the vascular area of interest, with segmentation failure that could not be manually corrected, and with poor-quality images or a poor signal strength were excluded (Figure 1, Figure 2 and Figure 3).

The current A.I. software was uploaded to the OCT machine.

The newest A.I. DeNoise™ technology improves tomogram quality powered by A.I. Advanced A.I. algorithms enhance the quality of a single tomogram to the level of an averaged tomogram obtained through multiple scans. The A.I. DeNoise™ algorithm filters out noise from the tomogram for the highest and smoothest image quality. The function is available on all tomograms. The moment a tomogram is loaded for review, the software starts denoising it. After a short moment, the original “undenoised” tomogram is replaced with a noise-free image obtaining a better visualization.

The A.I. Retina is a new layer segmentation for the posterior segment that is based on artificial intelligence, resulting in more accurate recognition of retinal layer boundaries.

The maximum intensity projection (MIP) algorithm provides better visualization of OCTA data for analysis. This MIP tool is useful for visualizing OCTA data, as it enables easier identification and tracking of high-intensity structures such as blood vessels.

The A.I. system has a direct impact on the accuracy of the clinical assessment and the assessment of the status of areas of pathology in the retina. This level of detection is more accurate and results in more detailed screening. Overall, it is a more effective way of running a pathology evaluation. A.I. segmentation is important for follow-up examinations, bringing a more accurate diagnosis when analyzing pathology over time.

### 2.3. Statistical Analysis

IBM Statistical Package for the Social Sciences (SPSS) V23, IBM SPSS Release 26.0.0.0, Minitab V16, JASP V 0.18.3, and Microsoft Office 2019 were used for statistical analysis.

The whole sample of 154 eyes was divided into two groups, with 77 subjects each: right eye and left eye, with each group subdivided into a hypercholesterolemia and hypertriglyceridemia group. Using descriptive statistics, the following indicators were calculated: mean, standard deviation (SD), mode, median, and 95% confidence intervals (CI). We analyzed age, sex, rural and urban areas, smoking habit, blood pressure (systolic and diastolic), cholesterol levels including total and HDL and LDL, triglycerides, BMI, glycemia, and HbA1c. For each group, the following parameters of the OCTA were determined for both eyes: FAZ area, FAZ perimeter, FAZ circularity, NFA area, VFA area, VFA flow area, density total, density ETDRS, skeleton total, and skeleton ETDRS.

The correlation between the blood pressure and the OCTA parameters was calculated using Pearson Correlation and Sig. (2-tailed) *p*-value test. Correlations between blood pressure, total cholesterol, HDL, LDL, triglycerides, and OCTA parameters were determined using Pearson correlation coefficient (r) and two-tailed *p*-value. We divided subjects with dyslipidemia into two groups, namely a hypercholesterolemia group (we analyzed total cholesterol, HDL, and LDL) and hypertriglyceridemia group (we analyzed triglycerides), and we determined correlation with OCTA parameters for the right eye and left eye.

A dependent *t*-test was used to determine if there is a significant difference between OCTA parameters in the right eye and the left-eye group, analyzing t-value (t), *p*-value (*p*), and degrees of freedom (df).

A multivariate analysis exploring how these factors interact collectively was performed. Factor analysis was performed using Principal component analysis of IBM SPSS Release 26.0.0.0. Kaiser–Meyer–Olkin test, Bartlett’s test, rotated component matrix, total variance explained, and scree plot were used.

## 3. Results

### 3.1. Descriptive Analysis

A total of 154 eyes were eligible for the final analysis presented in this study.

Glycemia and HbA1c were determined in order to rule out subjects with diabetes and/or undiagnosed diabetic retinopathy. Age, sex, smoking habit, and rural and urban areas were analyzed and are shown in Table 1.

The average age of the participants was 56.9 ± 9.1 years, with a minimum of 34 years and a maximum age of 82 years, and with a higher percentage of males: 55.8%. Overall, 31.2% subjects were recruited from rural areas and 68.8% subjects from urban areas; 27.3% were smokers, and 72.7% non-smokers.

The main clinical characteristics of the whole sample are shown in Table 1.

Table 2 shows the mean, standard deviation (SD), mode, median, and 95% confidence intervals (CI) mean calculated for blood pressure (SBP and DBP) and clinical variables in subjects with hypertension and dyslipidemia (Table S1).

**Figure 4:**
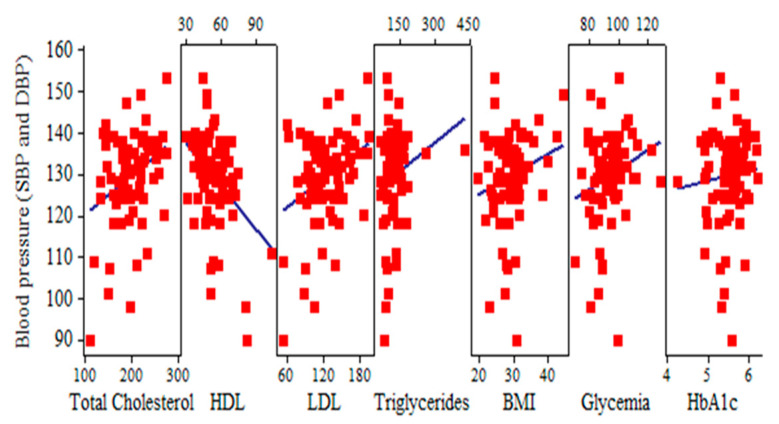
Statistical indicators of clinical variables.

Hypertensive participants with a higher mean SBP and DBP (129.5 ± 11.1, respectively 78.5 ± 9.6) had a significantly higher mean total cholesterol (197.7 ± 38.3) and mean LDL (124.0 ± 32.7) and a significantly higher mean BMI (28.8 ± 4.7). The mean HDL was 52.6 ± 13.3. Other clinical variables of the whole population study, which were not significant among subjects with hypertension and dyslipidemia, such as triglycerides, glycemia, and HbA1c, are shown in the Table 2 and Figure 4. Subjects with diabetes were excluded from this study.

### 3.2. Analysis of Ophthalmological Parameters

The analysis of ophthalmological parameters is shown in Table 3, Table 4, Table 5, Table 6, Table 7, Table 8, Table 9, Table 10, Table 11, Table 12 and Table 13.

Subjects were divided into two groups: right eye and left eye. For each group, the mean, median, mode, 95% CI mean, standard deviation (SD), and minimum and maximum were calculated and are shown in Table 3 and Table 4. Also, for each eye group, we determined OCTA parameters and correlations with hypercholesterolemia (Table 5 and Table 6) and with hypertriglyceridemia (Table 7 and Table 8).

Table 3 and Table 4 provide statistical indicators for the OCTA parameters for each eye group.

By analyzing the hypercholesterolemia in the right-eye group, we found the correlation between VFA area and HDL level to be statistically significant (r = 0.274; *p* = 0.016), showing that the higher the HDL level, the more the vascular flow is improved. Other results are also shown in Table 5 and are not of statistical significance.

In the left-eye group with hypercholesterolemia shown in Table 6, statistically significant results were determined for total cholesterol and FAZ circularity (r = 0.287; *p* = 0.011), NFA area (r = 0.245; *p* = 0.032), VFA area (r = 0.273; *p* = 0.016), VFA flow (r= −0.257; *p* = 0.024), skeleton total (r= −0.249; *p* = 0.029), and skeleton ETDRS (r= −0.254; *p* = 0.026).

The correlation between FAZ circularity and LDL (r = 0.313; *p* = 0.006), NFA area and LDL (r = 0.233; *p* = 0.042), and VFA area and LDL (r= −0.354; *p* = 0.002) were statistically significant. Higher values of skeleton total were correlated with higher values of HDL (r= 0.230; *p* = 0.044). This shows that by improving the HDL level, an improvement in the capillary circulation is obtained.

Our results show that subjects with higher values of LDL and total cholesterol lose more capillary vessels and have a higher non-flow area and poorer vascular flow; this results in the progression of hypertensive retinopathy in patients with poor dyslipidemia control.

No statistically significant correlations were found between hypertriglyceridemia and OCTA parameters in both eye groups. The results are shown in Table 7 and Table 8.

**Figure 5:**
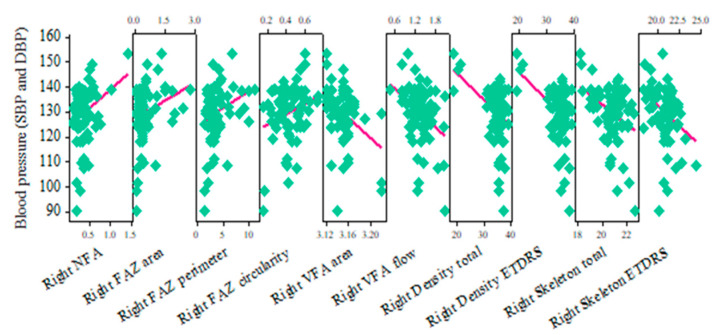
A matrix plot showing correlation of right-eye OCTA parameters with blood pressure.

By analyzing the right-eye group of hypertensive retinopathy shown in Table 9 and Figure 5, we found that four OCTA parameters were significantly higher in subjects with hypertension. The following results were obtained during our study for NFA area (r = 0.276; *p* = 0.015), FAZ area (r = 0.236; *p* = 0.039), FAZ perimeter (r = 0.253; *p* = 0.026), and FAZ circularity (r = 0.256; *p* = 0.025).

All other OCTA parameters, namely VFA area (r = −0.368; *p* = 0.001), VFA flow area (r = −0.364; *p* = 0.001), density total (r = −0.382; *p* = 0.001), density ETDRS (r = −0.399; *p* < 0.01), skeleton total (r = −0.364; *p* = 0.001), and skeleton ETDRS (r = −0.351; *p* = 0.002), were significantly lower in subjects with hypertension.

The results show avascular and non-flow areas increase in subjects with higher blood pressure, and a capillary drop-out as well as a decreased vascular flow are present in these cases. So, progression of hypertensive retinopathy in patients with higher blood pressure can be proven based on OCTA (Table S2).

**Figure 6:**
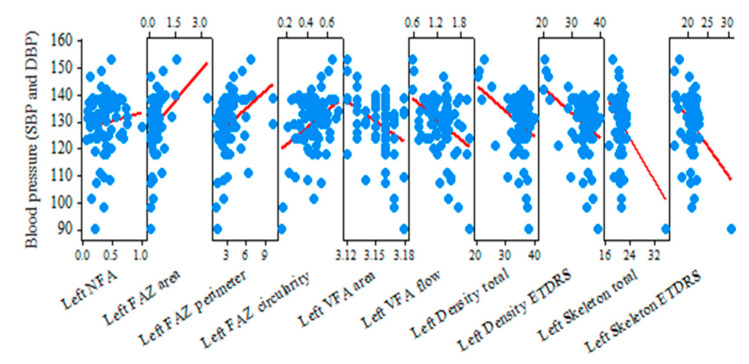
A matrix plot showing correlation of OCTA parameters in the left eye with blood pressure.

In the left-eye group of hypertensive retinopathy shown in Table 10 and Figure 6, the NFA area (r = 0.301; *p* = 0.008), FAZ area (r = 0.314; *p* = 0.005), FAZ perimeter (r = 0.322; *p* = 0.004), and FAZ circularity (r = 0.359; *p* = 0.001) values were also significantly higher in subjects with hypertension, just as with the right-eye group. All other OCTA parameters, namely VFA area (r = −0.373; *p* = 0.001), VFA flow area (r = −0.379; *p* = 0.001), density total (r = −0.345; *p* = 0.002), density ETDRS (r = −0.373; *p* = 0.001), skeleton total (r = −0.354; *p* = 0.002), and ETDRS (r = −0.363; *p* = 0.001), were lower when the blood pressure (SBP and DBP) was higher.

Patients with higher values of the blood pressure showed higher avascular zones, reduced blood flow, and a capillary drop-out. So, a progression of hypertensive retinopathy is demonstrated and measured with OCTA (Table S3).

The correlation between OCTA parameters was also calculated and shown in Table 9 and Table 10, using Pearson Correlation and Sig. (2-tailed) test and showing r-value and *p*-value. In subjects with a higher NFA area, the values of the FAZ area and perimeter were higher, while VFA area and flow area, density (total and ETDRS), and skeleton total were significantly lower.

In both groups, participants with higher values of density (total and ETDRS parameters) showed higher skeleton (total and ETDRS) levels.

Differences between right-eye OCTA parameters and left-eye OCTA parameters were also analyzed and are shown in Table 11 and Table 12.

A dependent 2-tailed *t*-test was used to determine if there is a significant difference between OCTA parameters in the right-eye group and OCTA parameters in the left-eye group, analyzing t-value (t), *p*-value (*p*), and degrees of freedom (df).

The *t*-test determined a value of *p* > 0.05, so no statistical significance was found between right-eye OCTA parameters and left-eye OCTA parameters. The highest values of *p* resulted for NFA area (*p* > 0.430), VFA area (*p* > 0.444), VFA flow (*p* > 0.564), density total (*p* > 0.838), and skeleton ETDRS (*p* > 0.810).

The MIP algorithm of the machine provides better visualization of OCTA data for analysis, as it enables easier identification and tracking of high-intensity structures such as blood vessels and more specific measurements of the OCTA parameters.

By associating the most relevant data obtained in our study, such as the statistically significant correlations calculated, the A.I. software can be improved by integrating new mathematic formulas to the current software to link OCTA parameters to blood pressure and lipids levels. Our most statistically significant correlations show that some OCTA variables, such as VFA area and flow, density total, and ETDRS and skeleton total, are inversely proportional to blood pressure, total cholesterol, and LDL, while other OCTA parameters, such as NFA area and FAZ area, perimeter, and circularity, are directly proportional to blood pressure, LDL, and total cholesterol.

In order to enhance the interpretation of the results, a multivariate analysis exploring how these factors interact collectively was performed using Kaiser–Meyer–Olkin (KMO) test, Bartlett’s test, rotated component matrix, total variance explained, and scree plot.

In order to demonstrate that the data used in correlations were reliable for factor analysis, a KMO and a Bartlett’s test were performed. The results are shown in Table 13 and Table 14. The KMO value in the right-eye group was 0.619 higher than the KMO value in the left eye, 0.531; thus, the data in right-eye group is more relevant.

Bartlett’s test resulted in a Sig. value < 0.01 in both eyes, showing that factor analysis was relevant in both groups.

The rotated component matrix is presented in Table 15 and Table 16. The rotation method used was Varimax with Kaiser normalization. For the right-eye group, five components were found to be relevant, and for the left-eye group, four relevant components were found.

The total variance explained is presented in Table 17 for both eyes. Values above 1 are statistically significant. In the right-eye group, five components with a cumulative % of 76.743% were found, while in the left-eye group, four components with a cumulative % value of 65.866% were obtained.

**Figure 7:**
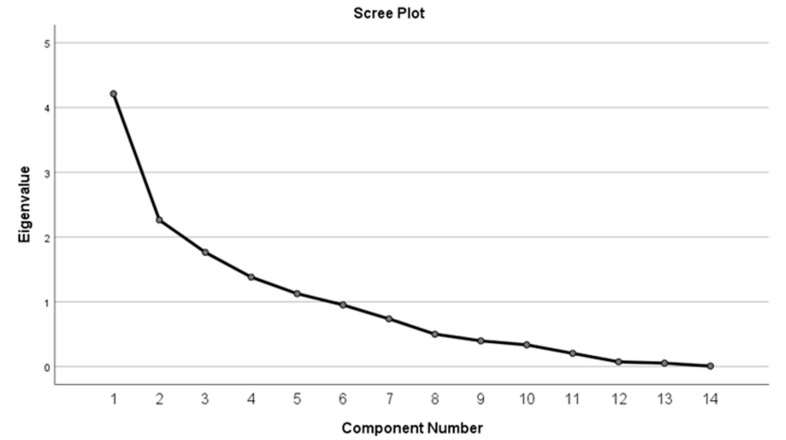
Scree plot—right eye.

**Figure 8:**
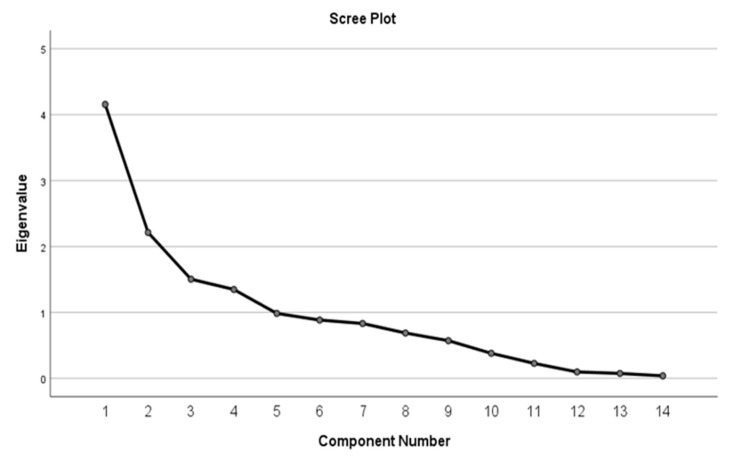
Scree plot—left eye.

The scree plot calculated for both eyes is shown in Figure 7 and Figure 8. Values above 1 are statistically significant.

## 4. Discussion

Hypertension is a very common condition worldwide and an important risk factor for cardiovascular and cerebrovascular events, renal diseases, and microvascular eye diseases [6]. Established hypertension leads to the chronic vasoconstriction of small arteries, impairment of the organ flow reserve, and the progressive worsening of hypertensive disease.

One of the latest studies about hypertensive retinopathy was published by Lei Jiao, Chaoran Lv, and Hongling Zhang [9]. This study concluded that hypertensive retinopathy is related to blood pressure variability (BPV), that the degree of BPV and retinopathy in hypertensive patients can be used to track the progression of hypertension-mediated organ damage (HOMD), and that the correction of BPV may help treat or postpone the progression of HOMD. Another study by Christopher Sun et al. showed that hypertension is associated with reduced retinal vessel density and an increased FAZ, especially in the deep venous plexus, as seen on OCTA [11].

In 2022, Niro, A et al. demonstrated in their study that patients with hypertensive retinopathy show a reduction in the retinal vascular density, superficial and deep vascular plexi (SVP and DVP), ganglion cell–internal boundary membrane complex density (GCL-ILM), and retinal nerve fiber layer (RNFL) thickness [12].

In the present study conducted on subjects with hypertension and dyslipidemia, the correlation between blood pressure (systolic and diastolic) and clinical variables showed that higher blood pressure is associated with higher total cholesterol and LDL and with lower HDL values. Triglycerides were not demonstrated to be statistically related to changes in OCTA parameters.

In our sample, participants with hypertension and a higher BMI and waist circumference had a higher blood pressure, suggesting a relationship between obesity and blood hypertension [19].

Our subjects had no diabetes and no undiagnosed diabetic retinopathy, and no relationship between hypertension and glycemia or HbA1c was found.

The relationship between blood pressure control, retinal blood vessels, choriocapillaris, and systemic vascular risk factors in hypertension was examined in our study.

We used the OCTA to confirm the association of blood pressure variability and dyslipidemia with OCTA parameters in hypertensive patients.

The choriocapillaris is composed of a dense network of capillaries, so it may be susceptible to damage as a result of uncontrolled systemic hypertension [6].

In the present study, we calculated the VFA (area and flow area parameters) at the level of the choriocapillaris. We reported a decrease in the capillary network in hypertensive subjects, which may contribute to flow reduction in and the impairment of oxygen delivery to different organs. The following values resulted for VFA area (r = −0.368; *p* = 0.001) and VFA flow area (r = −0.364; *p* = 0.001) in the right-eye group. In the left-eye group, VFA area (r = −0.373; *p* = 0.001) and VFA flow area (r = −0.379; *p* = 0.001) were obtained. These microvascular alterations may represent an early step in hypertension-mediated organ damage, and thus, they can be markers in monitoring the beneficial effects of antihypertensive treatment, according to Rizzoni D, Agabiti-Rosei C, Boari G.E.M., Muiesan ML, and De Ciuceis C. [10].

In our study conducted in a hypertensive retinopathy-based sample with OCTA performed in both eyes, higher NFA area and FAZ parameters (area, perimeter, and circularity) and lower VFA (area and flow area), density, and skeleton (total and ETDRS) were observed in hypertensive subjects.

In participants with higher FAZ area and perimeter, a reduction in VFA flow area, density, and skeleton was also observed. This shows that an increase in areas with no perfusion is associated with a decrease in blood flow and a decrease in perfused blood vessels.

Subjects with higher VFA (area and flow area) showed higher density and skeleton values, meaning that subjects with an increased area of blood vessels and an increased blood flow have a larger area of perfused vasculature.

Using the Pearson Correlation and Sig. (2-tailed) test, we discovered that higher density (total and ETDRS parameters) was closely connected with higher skeleton (total and ETDRS) in hypertensive individuals.

Analyzing both eyes groups with hypercholesterolemia, in the right-eye group, we found correlation between VFA area and HDL only to be statistically significant, while in the left-eye group, statistically significant correlations were determined for total cholesterol, HLD, LDL, and multiple OCTA parameters. Our results showed that higher values of total cholesterol and LDL were associated with increasing avascular areas, reduced vascular flow, and reduced vascular density.

In our study, no statistically significant correlations between hypertriglyceridemia and OCTA parameters in the right-eye and left-eye groups were found as well as for BP and hypertriglyceridemia.

We found no statistical significance between right-eye OCTA parameters and left-eye OCTA parameters in our study. The dependent *t*-test determined a value of *p* > 0.05.

OCTA is a more suitable technique for imaging the retinal microvasculature than the conventional invasive injectable dye angiography because OCTA allows for more efficient, rapid, non-invasive, and widely available imaging. Fluorescein angiography is unsuitable for use in large cohort studies and for consecutive follow-up visits. Compared to OCTA of the retina, no other widely available imaging modality has yet been able to measure microvasculature with such good resolution, reproducibility, and short examination time [20,21].

Furthermore, studying changes in the microvasculature on OCTA in relation to systemic vascular disease may have the potential to detect early disease, to monitor disease progression, to potentially provide a better staging of hypertensive retinopathy, and to detect improvements in vasculature in patients with a better response to treatment.

Patients with progressive retinopathy and dyslipidemia are susceptible to coronary artery disease, peripheral vascular disease, and cerebrovascular events due to similar damage in other end-organ systems [22].

Ong et al. noted in the Atherosclerosis Risk in Communities (ARIC) Study, which included 15,792 participants, that hypertensive retinopathy was associated with an increased risk of stroke, independent of other risk factors. They recommended the use of fundus examination to help assess the risk of stroke in these patients [23].

Chen and co-workers studied a number of 9793 hypertensive patients within the China Stroke Primary Prevention Trial. Of these, 592 patients suffered from a stroke at the end of a 4.4-year follow-up, and a significant association was found between the presence of hypertensive retinopathy and the risk of a cerebrovascular event [24]. Hua D, Xu Y, Zeng X, et al. noted that the microvascular structure of the retina and the cerebral and cardiac tissue have multiple similarities, so fundus examination may have an important role in the detection of end-organ damage. They suggested that the use of OCTA could increase the prediction of end-organ damage [4].

Additional damage to the eye is also possible. The persistence of arteriolar sclerosis can potentially lead to an increased incidence of arteriolar vein occlusions within the eye, as observed by Kotruchin P, Tangpaisarn T, Mitsungnern T, et al. [24].

Our findings were similar to the study performed by Chua et al. and by Sun et al. in that all our analyses confirmed a decrease in the deep capillary density in patients with systemic hypertension [6,11]. However, the difference with the study carried out by Sun et al. is that in our study did not determine intraocular pressure, visual acuity, and spherical equivalent, and our study did not compare the characteristics between groups, i.e., control and hypertensive. In addition, our study did not correlate choriocapillaris microvasculature with BP and renal parameters, which was analyzed by Chua et al. in their study. We had no control group, unlike other studies. Similar to the study performed by C Cuspidi et al. on non-diabetic hypertensive patients, retinal lesions were correlated with blood pressure, age, body mass index, smoking habit, and total serum cholesterol [7]. The difference is that we used OCTA as a tool for monitoring retinopathy progression, and we performed correlations between OCTA parameters and dyslipidemia.

In 2022, Rebecca Zeng et al. performed an observational study on retinal microvasculature and vasoreactivity changes in hypertension using OCTA [25]. Their study concluded that OCTA showed significant differences in the retinal vasculature of hypertensive patients, and it was also the first to demonstrate the potential of OCTA to investigate retinal vascular reactivity in patients with hypertension. Vessel density, vessel skeletonized density, and fractal dimension were calculated. A total of 23 eyes with hypertension (17 patients) and 17 control eyes (15 patients) were included, and a 6 mm × 6 mm angiogram centered on the fovea was performed. The superficial capillary plexus, vascular density, and the choriocapillaris were significantly decreased in hypertensive patients compared to control eyes. Our study had similar finding on microvascular changes, but we calculated the correlation between progressive retinopathy and microvascular changes in patients with dyslipidemia, with a larger number of subjects and without a control group. We did not study cerebral and cardiac tissue, like Hua D, Xu Y, Zeng X, et al. [26], and over a period of 18 months of monitoring our patients, we did not record any subjects with stroke, unlike Chen and co-workers [4].

A.I. computer systems are used extensively in medical sciences for diagnosing patients, discovering new drugs, improving communication with patients, and remotely treating patients [27]. In 2016, Gulshan V, Peng L, Coram M, Stumpe MC, Wu D, Narayanaswamy A, et al. developed and trained A.I. using 128,175 retinal fundus images to classify images as diabetic retinopathy and macular edema for adults with diabetes [28].

The new version of the Optopol OCT provides an A.I. algorithm for posterior-layer segmentations. By improving the current A.I. system and using OCTA findings, our research could provide new OCTA parameters for automated measurements, could help in early-stage detection of progressive hypertensive retinopathy, and could help develop screening programs, leading to increased efficiency in diagnosing patients in shorter time.

Therefore, based on our statistically significant results, we propose a few OCTA correlations to be analyzed using A.I., thus potentially resulting in new OCTA parameters: correlation between a lower VFA area and a lower HDL, a higher FAZ circularity with a higher LDL and total cholesterol, a higher LDL with a higher NFA area, and a lower skeleton total with a lower HDL.

However, there are a few limitations to this study. Firstly, we did not determine the values of apolipoprotein A1 and apolipoprotein B in correlation with OCTA parameters. Secondly, our changes in retinal vasculature were not correlated with renal function, heart function, other peripheral microvasculature parameters, or other end-organ damage measurements. Our current OCTA imaging (Angio-OCT Optopol Revo NX 130) depends on motion contrast from blood flow to visualize the retinal vasculature; hence, vessels with flow below the detectable threshold were not displayed. We acknowledge that our manual measurement performed in order to delimit FAZ, NFA, and VFA may not be as exact as the automated measurements performed by the OCTA, but this is unlikely to significantly affect our results. Also, our cohort only included 77 participants. Consecutive follow-up visits are required after our 18-month observational study.

We used the OCTA as a tool for monitoring retinopathy associated with dyslipidemia, but other complementary methods, such as fluorescein angiography, suitable for use in small cohort studies, and indocyanine green angiography (ICGA), which is another diagnostic procedure used to examine choroidal blood flow and associated pathology, should be required in order to obtain more precise results.

In our study, our primary objective was to use OCTA as a tool for analyzing the progression of hypertensive retinopathy in patients with dyslipidemia. Our secondary objective was to suggest that an improvement of the current A.I. system of the OCTA can be made.

The current A.I. system has a direct impact on the accuracy of the clinical assessment and the assessment of the status of areas of pathology in the retina. This level of detection is based on the A.I. Retina layer segmentation and MIP algorithm, thus resulting in more accurate results and more detailed screening. A.I. segmentation is important for follow-up examinations, bringing a more accurate diagnosis when analyzing pathology over time. The MIP tool enables easier identification and tracking of blood vessels. We suggest updating the current A.I system by introducing mathematic formulas in the system to show that some OCTA parameters, lipid levels, and blood pressure are directly proportional to each other, while some OCTA variables are inversely proportional to blood pressure and lipid levels, thus resulting in a more accurate evaluation and monitoring of retinal pathology in patients with dyslipidemia and hypertension.

## 5. Conclusions

In conclusion, the present findings confirmed the correlation between hypertensive retinopathy, dyslipidemia, and OCTA parameters, and apart from blood pressure reduction, targeting the microcirculation itself could be beneficial in preventing or reducing end-organ damage and thus reducing mortality and morbidity.

OCTA is a useful tool in detecting in detail the various alterations in retinal microvasculature [14,17,21,25,29]. Earlier detection upon OCTA of the small blood vessel changes, prior to the appearance of changes on the fundus examination, can provide a more accurate staging of the hypertensive retinopathy and can help us to better understand the pathophysiology involved.

Further studies, particularly with a lager cohort, are needed to test the effectiveness of retinal capillary density as a novel biomarker in predicting the incidence and progression of hypertension-related microvascular complications.

## Figures and Tables

**Table 1:** Characteristics of the patient sample.

Parameters		Frequency	Valid Percent (%)	Age (Years)	
				Mean ± SD	Median (Min to Max)
Sex	Females	34	44.2	58.8 ± 8.8	58.0 (35 to 79)
	Males	43	55.8	55.4 ± 9.1	55.0 (34 to 82)
Areas	Rural	24	31.2	56.0 ± 8.5	55.5 (34 to 71)
	Urban	53	68.8	57.3 ± 9.4	56.0 (35 to 82)
Smokers	Yes	21	27.3	56.0 ± 8.5	55.5 (34 to 71)
	No	56	72.7	57.3 ± 9.4	56.0 (35 to 82)
Total		77	100.0	56.9 ± 9.1	56.0 (34 to 82)

**Table 2:** Statistical indicators calculated for clinical variables.

Clinical Variables	Statistical Indicators			
	Mean ± SD	Median (Min to Max)	Mode	95% CI Mean (Lower to Upper)
SBP (mmHg)	129.5 ± 11.1	131.0 (90.0 to 153.0)	138.0	(127.0 to 132.0)
DBP (mmHg)	78.5 ± 9.6	78.0 (43.0 to 108.0)	74.0	(76.4 to 80.7)
Total Cholesterol (mg/dL)	197.7 ± 38.3	197.1 (109.3 to 275.6)	234.0	(189.0 to 206.4)
HDL (mg/dL)	52.6 ± 13.3	50.4 (29.3 to 103.4)	60.8	(49.5 to 55.6)
LDL (mg/dL)	124.0 ± 32.7	121.4 (53.4 to 194.6)	53.4	(116.6 to 131.4)
Triglycerides (mg/dL)	122.7 ± 48.8	112.4 (65.2 to 427.4)	95.8	(111.6 to 133.8)
BMI (kg/m^2^)	28.8 ± 4.7	28.4 (19.2 to 44.5)	25.1	(27.7 to 29.9)
Glycemia (mg/dL)	92.7 ± 10.4	92.2 (69.7 to 128.3)	95.5	(90.4 to 95.1)
HbA1c (%)	5.5 ± 0.4	5.5 (4.2 to 6.2)	5.9	(5.4 to 5.6)

SBP: systolic blood pressure; DBP diastolic blood pressure; HDL: high-density cholesterol, LDL: low-density cholesterol; BMI: body mass index; HbA1c: glycated hemoglobin. All data are shown as mean ± standard deviation (SD), median (min to max) for continuous variables, mode, and 95% confidence interval mean (lower to upper).

**Table 3:** The mean, median, mode, 95% CI mean, standard deviation (SD), and minimum and maximum for ophthalmological parameters in the right-eye group.

OCTA Right Eye	Statistical Indicators			
	Mean ± SD	Median (Min to Max)	Mode	95% CI Mean (Lower to Upper)
NFA Area (mm^2^)	0.4 ± 0.2	0.3 (0.1 to 1.5)	0.4	(0.3 to 0.4)
FAZ Area (mm^2^)	0.5 ± 0.6	0.4 (0.1 to 2.8)	0.5	(0.4 to 0.7)
FAZ Perimeter (mm)	3.7 ± 2.1	2.9 (1.3 to 11.4)	3.7	(3.2 to 4.2)
FAZ Circularity	0.4 ± 0.1	0.4 (0.2 to 0.8)	0.4	(0.4 to 0.5)
VFA Area (mm^2^)	3.2 ± 0.0	3.2 (3.1 to 3.2)	3.2	(3.1 to 3.2)
VFA Flow (mm^2^)	1.3 ± 0.3	1.3 (0.4 to 2.1)	1.3	(1.3 to 1.4)
Density Total	34.5 ± 4.0	35.5 (18.3 to 39.0)	34.5	(33.6 to 35.4)
Density ETDRS	34.4 ± 4.0	35.3 (18.9 to 39.1)	34.4	(33.5 to 35.3)
Skeleton Total	20.8 ± 1.1	20.8 (18.1 to 22.7)	20.8	(20.5 to 21.0)
Skeleton ETDRS	20.8 ± 1.2	20.9 (18.2 to 24.4)	20.8	(20.5 to 21.0)

FAZ: foveal avascular zone; NFA: non-flow area; VFA: vascular flow area; ETDRS: Early Treatment of Diabetic Retinopathy Study. All data are shown as mean ± standard deviation (SD), median (min to max) for continuous variables, mode, and 95% confidence interval mean (lower to upper).

**Table 4:** Statistical indicators for the left-eye group.

OCTA Left Eye	Statistical Indicators			
	Mean ± SD	Median (Min to Max)	Mode	95% CI Mean (Lower to Upper)
NFA Area (mm^2^)	0.4 ± 0.2	0.4 (0.1 to 0.8)	0.2	(0.3 to 0.4)
FAZ Area (mm^2^)	0.4 ± 0.5	0.3 (0.1 to 3.5)	0.2	(0.3 to 0.5)
FAZ Perimeter (mm)	3.5 ± 1.7	3.1 (1.4 to 10.3)	2.3	(3.1 to 3.9)
FAZ Circularity	0.5 ± 0.1	0.5 (0.2 to 0.7)	0.5	(0.4 to 0.5)
VFA Area (mm^2^)	3.2 ± 0.0	3.2 (3.1 to 3.2)	3.2	(3.1 to 3.2)
VFA Flow (mm^2^)	1.3 ± 0.3	1.4 (0.6 to 2.0)	1.4	(1.2 to 1.4)
Density Total	34.6 ± 4.0	35.6 (20.3 to 40.1)	34.5	(33.7 to 35.5)
Density ETDRS	33.9 ± 4.4	34.7 (19.8 to 40.0)	33.4	(32.9 to 34.9)
Skeleton Total	20.9 ± 2.0	20.9 (16.9 to 35.7)	21.0	(20.5 to 21.4)
Skeleton ETDRS	20.7 ± 1.9	20.9 (16.3 to 30.7)	20.4	(20.3 to 21.1)

FAZ: foveal avascular zone; NFA: non-flow area; VFA: vascular flow area; ETDRS: Early Treatment of Diabetic Retinopathy Study. All data are shown as mean ± standard deviation (SD), median (min to max) for continuous variables, mode, and 95% confidence interval mean (lower to upper).

**Table 5:** Right-eye group with hypercholesterolemia and correlations with OCTA parameters.

Correlations				
OCTA Parameters: Right Eye	Pearson Correlation	Total Cholesterol	HDL	LDL
FAZ Area	Pearson Correlation	0.020	−0.091	−0.108
	Sig. (2-tailed)	0.864	0.431	0.350
FAZ Perimeter	Pearson Correlation	0.056	−0.079	−0.058
	Sig. (2-tailed)	0.630	0.495	0.615
FAZ Circularity	Pearson Correlation	0.198	−0.053	0.216
	Sig. (2-tailed)	0.085	0.645	0.059
NFA Area	Pearson Correlation	0.126	−0.103	0.016
	Sig. (2-tailed)	0.275	0.371	0.893
VFA Area	Pearson Correlation	0.016	0.274 *	0.023
	Sig. (2-tailed)	0.891	0.016	0.841
VFA Flow	Pearson Correlation	−0.160	0.050	−0.056
	Sig. (2-tailed)	0.163	0.667	0.628
Density Total	Pearson Correlation	−0.124	0.148	−0.145
	Sig. (2-tailed)	0.282	0.198	0.209
Density ETDRS	Pearson Correlation	−0.136	0.149	−0.149
	Sig. (2-tailed)	0.238	0.194	0.197
Skeleton Total	Pearson Correlation	−0.125	0.198	−0.108
	Sig. (2-tailed)	0.278	0.084	0.348
Skeleton ETDRS	Pearson Correlation	0.037	0.143	0.076
	Sig. (2-tailed)	0.750	0.214	0.511

FAZ: foveal avascular zone; NFA: non-flow area; VFA: vascular flow area; ETDRS: Early Treatment of Diabetic Retinopathy Study: right eye; HDL: high-density cholesterol, LDL: low-density cholesterol. * Correlation is significant at the 0.05 level (2-tailed).

**Table 6:** Left-eye group with hypercholesterolemia and correlations with OCTA parameters.

Correlations				
OCTA Parameters: Left Eye	Pearson Correlation	Total cholesterol	HDL	LDL
FAZ Area	Pearson Correlation	0.167	−0.141	−0.066
	Sig. (2-tailed)	0.146	0.220	0.569
FAZ Perimeter	Pearson Correlation	0.099	−0.082	−0.033
	Sig. (2-tailed)	0.393	0.477	0.777
FAZ Circularity	Pearson Correlation	0.287 *	−0.178	0.313 **
	Sig. (2-tailed)	0.011	0.121	0.006
NFA Area	Pearson Correlation	0.245 *	−0.025	0.232 *
	Sig. (2-tailed)	0.032	0.826	0.042
VFA Area	Pearson Correlation	−0.273 *	0.018	−0.354 **
	Sig. (2-tailed)	0.016	0.878	0.002
VFA Flow	Pearson Correlation	−0.257 *	−0.021	−0.142
	Sig. (2-tailed)	0.024	0.856	0.218
Density Total	Pearson Correlation	−0.112	0.119	−0.131
	Sig. (2-tailed)	0.332	0.305	0.256
Density ETDRS	Pearson Correlation	−0.078	0.100	−0.042
	Sig. (2-tailed)	0.501	0.385	0.716
Skeleton Total	Pearson Correlation	−0.249 *	0.230 *	−0.178
	Sig. (2-tailed)	0.029	0.044	0.120
Skeleton ETDRS	Pearson Correlation	−0.254 *	0.186	−0.177
	Sig. (2-tailed)	0.026	0.105	0.124

FAZ: foveal avascular zone; NFA: non-flow area; VFA: vascular flow area; ETDRS: Early Treatment of Diabetic Retinopathy Study: left eye; HDL: high-density cholesterol, LDL: low-density cholesterol. * Correlation is significant at the 0.05 level (2-tailed). ** Correlation is significant at the 0.01 level (2-tailed).

**Table 7:** Right-eye group with hypertriglyceridemia. Correlations with OCTA parameters.

Correlations		
OCTA Parameters	Pearson Correlation	Triglycerides
FAZ Area	Pearson Correlation	−0.092
	Sig. (2-tailed)	0.425
FAZ Perimeter	Pearson Correlation	−0.076
	Sig. (2-tailed)	0.511
FAZ Circularity	Pearson Correlation	0.058
	Sig. (2-tailed)	0.615
NFA Area	Pearson Correlation	−0.139
	Sig. (2-tailed)	0.227
VFA Area	Pearson Correlation	−0.189
	Sig. (2-tailed)	0.099
VFA Flow	Pearson Correlation	0.090
	Sig. (2-tailed)	0.437
Density Total	Pearson Correlation	0.033
	Sig. (2-tailed)	0.773
Density ETDRS	Pearson Correlation	0.036
	Sig. (2-tailed)	0.753
Skeleton Total	Pearson Correlation	−0.113
	Sig. (2-tailed)	0.326
Skeleton ETDRS	Pearson Correlation	−0.168
	Sig. (2-tailed)	0.144

FAZ: foveal avascular zone; NFA: non-flow area; VFA: vascular flow area; ETDRS: Early Treatment of Diabetic Retinopathy Study: right eye.

**Table 8:** Left-eye group with hypertriglyceridemia. Correlations with OCTA parameters.

Correlations		
OCTA Parameters	Pearson Correlation	Triglycerides
FAZ Area	Pearson Correlation	−0.068
	Sig. (2-tailed)	0.557
FAZ Perimeter	Pearson Correlation	−0.073
	Sig. (2-tailed)	0.527
FAZ Circularity	Pearson Correlation	0.071
	Sig. (2-tailed)	0.542
NFA Area	Pearson Correlation	−0.072
	Sig. (2-tailed)	0.534
VFA Area	Pearson Correlation	−0.103
	Sig. (2-tailed)	0.371
VFA Flow	Pearson Correlation	−0.048
	Sig. (2-tailed)	0.679
Density Total	Pearson Correlation	0.116
	Sig. (2-tailed)	0.315
Density ETDRS	Pearson Correlation	0.083
	Sig. (2-tailed)	0.473
Skeleton Total	Pearson Correlation	−0.053
	Sig. (2-tailed)	0.650
Skeleton ETDRS	Pearson Correlation	−0.001
	Sig. (2-tailed)	0.990

FAZ: foveal avascular zone; NFA: non-flow area; VFA: vascular flow area; ETDRS: Early Treatment of Diabetic Retinopathy Study: left eye.

**Table 9:** The ophthalmological parameters in the right-eye group with hypertensive retinopathy and the correlation with SBP and DBP, calculated using Pearson Correlation and Sig. (2-tailed) test.

RIGHT EYE OCTA		RIGHT EYE OCTA										
		SBP and DBP	NFA Area	FAZ Area	FAZ Perimeter	FAZ Circularity	VFA Area	VFA Flow Area	Density Total	Density ETDRS	Skeleton Total	Skeleton ETDRS
SBP and DBP	Pearson Correlation	1	0.276 *	0.236 *	0.253 *	0.256 *	−0.368 **	−0.364 **	−0.382 **	−0.399 **	−0.364 **	−0.351 **
	Sig. (2-tailed)		0.015	0.039	0.026	0.025	0.001	0.001	0.001	0.000	0.001	0.002
NFA Area	Pearson Correlation	0.276 *	1	0.512 **	0.491 **	0.112	−0.289 *	−0.242 *	−0.433 **	−0.449 **	−0.382 **	−0.103
	Sig. (2-tailed)	0.015		0.000	0.000	0.333	0.011	0.034	0.000	0.000	0.001	0.371
FAZ Area	Pearson Correlation	0.236 *	0.512 **	1	0.927 **	−0.253 *	−0.302 **	−0.281 *	−0.268 *	−0.320 **	−0.393 **	−0.246 *
	Sig. (2-tailed)	0.039	0.000		0.000	0.026	0.008	0.013	0.018	0.005	0.000	0.031
FAZ Perimeter	Pearson Correlation	0.253 *	0.491 **	0.927 **	1	−0.374 **	−0.255 *	−0.322 **	−00.211	−0.269 *	−0.348 **	−0.235 *
	Sig. (2-tailed)	0.026	0.000	0.000		0.001	0.025	0.004	0.065	0.018	0.002	0.040
FAZ Circularity	Pearson Correlation	0.256 *	0.112	−0.253 *	−0.374 **	1	−0.137	−0.085	−0.125	−0.109	−0.078	0.088
	Sig. (2-tailed)	0.025	0.333	0.026	0.001		0.234	0.461	0.277	0.347	0.499	0.445
VFA Area	Pearson Correlation	−0.368 **	−0.289 *	−0.302 **	−0.255 *	−0.137	1	0.039	0.230 *	0.267 *	0.397 **	0.279 *
	Sig. (2-tailed)	0.001	0.011	0.008	0.025	0.234		0.739	0.044	0.019	0.000	0.014
VFA Flow Area	Pearson Correlation	−0.364 **	−0.242 *	−0.281 *	−0.322 **	−0.085	0.039	1	0.145	0.142	0.221	0.253 *
	Sig. (2-tailed)	0.001	0.034	0.013	0.004	0.461	0.739		0.209	0.218	0.053	0.026
Density Total	Pearson Correlation	−0.382 **	−0.433 **	−0.268 *	−0.211	−0.125	0.230 *	0.145	1	0.989 **	0.775 **	0.472 **
	Sig. (2-tailed)	0.001	0.000	0.018	0.065	0.277	0.044	0.209		0.000	0.000	0.000
Density ETDRS	Pearson Correlation	−0.399 **	−0.449 **	−0.320 **	−0.269 *	−0.109	0.267 *	0.142	0.989 **	1	0.788 **	0.490 **
	Sig. (2-tailed)	0.000	0.000	0.005	0.018	0.347	0.019	0.218	0.000		0.000	0.000
Skeleton Total	Pearson Correlation	−0.364 **	−0.382 **	−0.393 **	−0.348 **	−0.078	0.397 **	0.221	0.775 **	0.788 **	1	0.613 **
	Sig. (2-tailed)	0.001	0.001	0.000	0.002	0.499	0.000	0.053	0.000	0.000		0.000
Skeleton ETDRS	Pearson Correlation	−0.351 **	−0.103	−0.246 *	−0.235 *	0.088	0.279 *	0.253 *	0.472 **	0.490 **	0.613 **	1
	Sig. (2-tailed)	0.002	0.371	0.031	0.040	0.445	0.014	0.026	0.000	0.000	0.000	

SBP: systolic blood pressure; DBP: diastolic blood pressure; FAZ: foveal avascular zone; NFA: non-flow area; VFA: vascular flow area; ETDRS: Early Treatment of Diabetic Retinopathy Study. * Correlation is significant at the 0.05 level (2-tailed). ** Correlation is significant at the 0.01 level (2-tailed).

**Table 10:** OCTA parameters in the left-eye group with hypertensive retinopathy. Correlation with blood pressure and between OCTA parameters, also calculated using Pearson Correlation and Sig. (2-tailed) test.

LEFT EYE OCTA		LEFT EYE OCTA										
		SBP and DBP	NFA Area	FAZ Area	FAZ Perimeter	FAZ Circularity	VFA Area	VFA Flow Area	Density Total	Density ETDRS	Skeleton Total	Skeleton ETDRS
SBP and DBP	Pearson Correlation	1	0.301 **	0.314 **	0.322 **	0.359 **	−0.373 **	−0.379 **	−0.345 **	−0.373 **	−0.354 **	−0.363 **
	Sig. (2-tailed)		0.008	0.005	0.004	0.001	0.001	0.001	0.002	0.001	0.002	0.001
NFA Area	Pearson Correlation	0.301 **	1	0.385 **	0.338 **	0.171	−0.1519	−0.19888	−0.287 *	−0.330 **	−0.306 **	−0.424 **
	Sig. (2-tailed)	0.008		0.001	0.003	0.136	0.187	0.083	0.011	0.003	0.007	0.000
FAZ Area	Pearson Correlation	0.314 **	0.385 **	1	0.726 **	0.02113677	0.03426	−0.342 **	−0.240 *	−0.273 *	−0.280 *	−0.394 **
	Sig. (2-tailed)	0.005	0.001		0.000	0.855	0.767	0.002	0.036	0.016	0.014	0.000
FAZ Perimeter	Pearson Correlation	0.322 **	0.338 **	0.726 **	1	−0.226 *	−0.0318	−0.293 **	−0.297 **	−0.326 **	−0.270 *	−0.355 **
	Sig. (2-tailed)	0.004	0.003	0.000		0.048	0.784	0.010	0.009	0.004	0.018	0.002
FAZ Circularity	Pearson Correlation	0.359 **	0.171	0.021	−0.226 *	1	−0.160	−0.263 *	−0.252 *	−0.196	−0.198	−0.082
	Sig. (2-tailed)	0.001	0.136	0.855	0.048		0.165	0.021	0.027	0.088	0.085	0.476
VFA Area	Pearson Correlation	−0.373 **	−0.152	0.034	−0.032	−0.160	1	0.157	0.322 **	0.239 *	0.239 *	0.239 *
	Sig. (2-tailed)	0.001	0.187	0.767	0.784	0.165		0.172	0.004	0.036	0.036	0.036
VFA Flow Area	Pearson Correlation	−0.379 **	−0.199	−0.342 **	−0.293 **	−0.263 *	0.157	1	0.195	0.343 **	0.315 **	0.366 **
	Sig. (2-tailed)	0.001	0.083	0.002	0.010	0.021	0.172		0.089	0.002	0.005	0.001
Density Total	Pearson Correlation	−0.345 **	−0.287 *	−0.240 *	−0.297 **	−0.252 *	0.322 **	0.195	1	0.887 **	0.386 **	0.482 **
	Sig. (2-tailed)	0.002	0.011	0.036	0.009	0.027	0.004	0.089		0.000	0.001	0.000
Density ETDRS	Pearson Correlation	−0.373 **	−0.330 **	−0.273 *	−0.326 **	−0.196	0.239 *	0.343 **	0.887 **	1	0.441 **	0.539 **
	Sig. (2-tailed)	0.001	0.003	0.016	0.004	0.088	0.036	0.002	0.000		0.000	0.000
Skeleton Total	Pearson Correlation	−0.354 **	−0.306 **	−0.280 *	−0.270 *	−0.198	0.239 *	0.315 **	0.386 **	0.441 **	1	0.870 **
	Sig. (2-tailed)	0.002	0.007	0.014	0.018	0.085	0.036	0.005	0.001	0.000		0.000
Skeleton ETDRS	Pearson Correlation	−0.363 **	−0.424 **	−0.394 **	−0.355 **	−0.082	0.239 *	0.366 **	0.482 **	0.539 **	0.870 **	1
	Sig. (2-tailed)	0.001	0.000	0.000	0.002	0.476	0.036	0.001	0.000	0.000	0.000	

* Correlation is significant at the 0.05 level (2-tailed). ** Correlation is significant at the 0.01 level (2-tailed).

**Table 11:** The difference between right-eye and left-eye OCTA parameters. The mean, median, mode, 95% CI mean, standard deviation (SD), and minimum and maximum were calculated.

OCTA Parameters	Mean	Median	Mode	95% CI Mean		SD	Min.	Max.
				Lower	Upper			
NFA Area	0.02	0.01	0.08	−0.03	0.07	0.21	−0.53	0.68
FAZ Area	0.10	0.00	0.00	−0.01	0.21	0.48	−0.86	1.85
FAZ Perimeter	0.19	−0.03	0.00	−0.22	0.60	1.82	−3.27	6.97
FAZ Circularity	−0.02	0.00	0.00	−0.06	0.01	0.15	−0.39	0.28
VFA Area	0.00	0.00	0.00	−0.01	0.00	0.02	−0.06	0.09
VFA Flow	0.03	−0.04	0.19	−0.06	0.11	0.35	−0.85	0.98
Density Total	−0.07	−0.40	−0.40	−0.70	0.57	2.78	−4.40	10.80
Density ETDRS	0.45	0.00	−1.00	−0.31	1.22	3.37	−6.10	15.70
Skeleton Total	−0.17	−0.10	−1.10	−0.60	0.26	1.88	−13.00	4.00
Skeleton ETDRS	0.06	−0.10	−1.20	−0.40	0.50	1.98	−10.60	4.70

FAZ: foveal avascular zone; NFA: non-flow area; VFA: vascular flow area; ETDRS: Early Treatment of Diabetic Retinopathy Study; SD: standard deviation.

**Table 12:** The dependent 2-tailed *t*-test comparing right-eye and left-eye OCTA parameters.

OCTA Right Eye	OCTA Left Eye	t	df	*p*
NFA Area	NFA Area	0.793	76	0.430
FAZ Area	FAZ Area	1.810	76	0.074
FAZ Perimeter	FAZ Perimeter	0.913	76	0.364
FAZ Circularity	FAZ Circularity	1.322	76	0.190
VFA Area	VFA Area	0.770	76	0.444
VFA Flow	VFA Flow	0.579	76	0.564
Density Total	Density Total	0.205	76	0.838
Density ETDRS	Density ETDRS	1.181	76	0.241
Skeleton Total	Skeleton Total	0.788	76	0.433
Skeleton ETDRS	Skeleton ETDRS	0.242	76	0.810

FAZ: foveal avascular zone; NFA: non-flow area; VFA: vascular flow area; ETDRS: Early Treatment of Diabetic Retinopathy Study; t: t-value, df: degrees of freedom; *p*: *p*-value.

**Table 13:** Kaiser–Meyer–Olkin and Bartlett’s test in the right-eye group.

Kaiser–Meyer–Olkin Measure of Sampling Adequacy.		**0.619**
Bartlett’s Test of Sphericity	Approx. Chi-Square	834.421
	df	91
	Sig.	**<0.001**

**Table 14:** Kaiser–Meyer–Olkin and Bartlett’s test in the left-eye group.

Kaiser–Meyer–Olkin Measure of Sampling Adequacy.		**0.531**
Bartlett’s Test of Sphericity	Approx. Chi-Square	631.589
	df	91
	Sig.	**<0.001**

**Table 15:** Rotated component matrix in the right-eye group.

	Component				
	1	2	3	4	5
Total Density	0.928	−0.171			−0.164
ETDRS density	0.925	−0.217			−0.139
Total Skeleton	0.847	−0.262		0.212	
Skeleton ETDRS	0.699			0.247	0.321
FAZ Perimeter	−0.124	0.873			−0.390
FAZ Area	−0.173	0.870			−0.287
NFA Area	−0.264	0.724			0.338
VFA Flow	0.106	−0.478	−0.125		
Total Cholesterol		0.125	0.966		
LDL			0.931		0.127
Triglycerides		−0.240	0.366	−0.750	−0.198
HDL	0.104		0.258	0.727	−0.119
VFA Area	0.214	−0.327		0.601	−0.188
FAZ Circularity		−0.117	0.176	−0.126	0.858

Extraction method: principal component analysis. Rotation method: Varimax with Kaiser normalization. Rotation converged in five iterations.

**Table 16:** Rotated component matrix in the left-eye group.

	Component			
	1	2	3	4
Total Density	0.876	−0.177		
Density ETDRS	0.859	−0.270		
Skeleton ETDRS	0.588	−0.542	−0.1424	0.211
Skeleton Total	0.558	−0.418	−0.169	0.322
VFA Area	0.435		−0.415	
FAZ Circularity	−0.402	−0.195	0.397	−0.244
FAZ Area		0.870		
FAZ Perimeter		0.869	−0.114	
NFA Area	−0.284	0.508	0.233	
VFA Flow	0.247	−0.441	−0.254	
Total Cholesterol		0.232	0.930	
LDL			0.927	
HDL	0.189		0.283	0.811
Triglycerides	0.191		0.289	−0.734

Extraction method: principal component analysis. Rotation method: Varimax with Kaiser normalization. Rotation converged in five iterations.

**Table 17:** Total variance explained—right eye and left eye.

Component	Extraction Sums of Squared Loadings			Rotation Sums of Squared Loadings		
	Total	% of Variance	Cumulative %	Total	% of Variance	Cumulative %
1	4.211	30.081	30.081	3.116	22.256	22.256
2	2.262	16.160	46.241	2.614	18.669	40.925
3	1.764	12.597	58.838	2.071	14.794	55.719
4	1.381	9.866	68.704	1.595	11.391	67.110
5	1.125	8.039	76.743	1.349	9.633	76.743
1	4.156	29.683	29.683	2.737	19.552	19.552
2	2.213	15.807	45.490	2.640	18.858	38.410
3	1.505	10.747	56.236	2.405	17.178	55.588
4	1.348	9.629	65.866	1.439	10.278	65.866

Extraction method: principal component analysis.

## Data Availability

The data that support the findings of the present study are available from the corresponding author (G.S.) upon reasonable request.

## Supplementary Materials

The following supporting information can be downloaded at https://www.mdpi.com/article/10.3390/jcm13247584/s1, Table S1. Matrix Plot. Correlation between blood pressure (systolic and diastolic) and clinical variables; Table S2. Matrix Plot. Correlation between blood pressure (systolic and diastolic) and Right-Eye OCTA parameters; Table S3. Matrix Plot. Correlation between blood pressure (systolic and diastolic) and Left-Eye OCTA parameters.
