# Supplementary material for: Monitoring Progression in Hypertensive Patients with Dyslipidemia Using Optical Coherence Tomography Angiography: Can A.I. Be Improved?
Source: J Clin Med. 2024 Dec 13;13(24):7584. doi: 10.3390/jcm13247584 (PMC11678628; doi:10.3390/jcm13247584)

Table S1. Matrix Plot. Correlation between blood pressure (systolic and diastolic) and clinical variables.

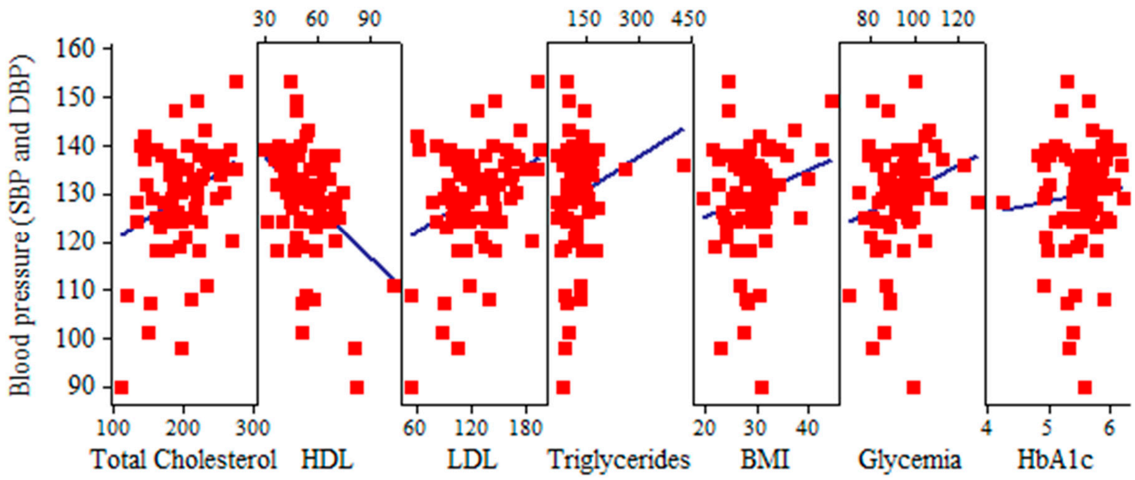

Table S2. Matrix Plot. Correlation between blood pressure (systolic and diastolic) and Right Eye OCTA parameters.

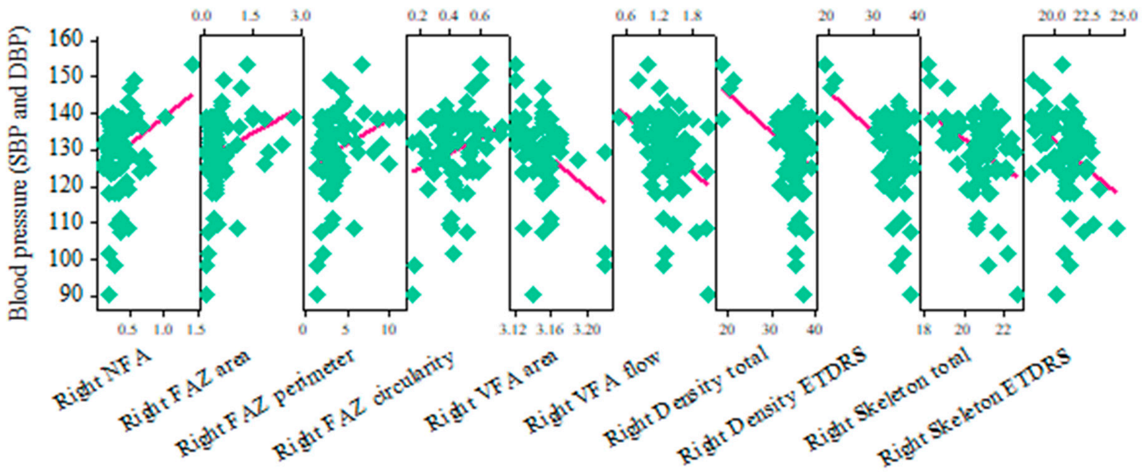

Table S3. Matrix Plot. Correlation between blood pressure (systolic and diastolic) and Left Eye OCTA parameters.

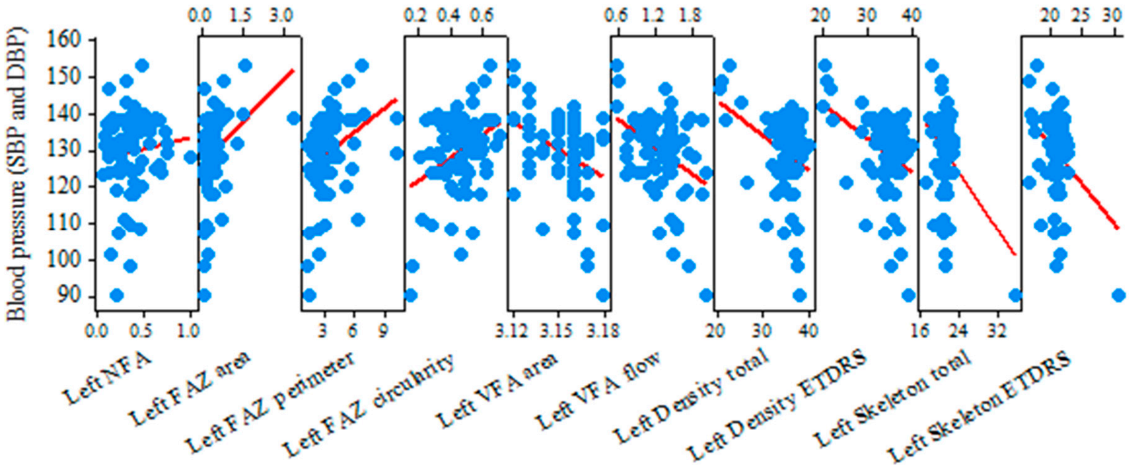

Supplement: Supplementary file 1 [file jcm-13-07584-s001.zip › jcm-3244718-supplementary.pdf]
